# The complete mitochondrial genome of the cowpea weevil, *Callosobruchus maculates* (Coleoptera: Chrysomelidae: Bruchinae) and a related phylogenetic analysis of Chrysomelidae

**DOI:** 10.1080/23802359.2017.1413308

**Published:** 2018-05-26

**Authors:** Li-Jie Zhang, Ling Wu, Chun-Yan Wei, Xing-Liang Liu, Huai-Jun Xue, Xing-Ke Yang, Rui-E Nie

**Affiliations:** a Inspection and Quarantine Technical Center, Beijing Inspection and Quarantine Testing Bureau, Beijing, China;; b Key Laboratory of Zoological Systematics and Evolution, Institute of Zoology, Chinese Academy of Sciences, Beijing, China;; c Jilin Entry-Exit Inspection and Quarantine Bureau, Changchun, China

**Keywords:** Mitochondrial genome, phylogeny, *Callosobruchus maculates*, Chrysomelidae

## Abstract

In this study, the complete 17,809 bp mitochondrial genome of *Callosobruchus maculates* (F.) (Coleoptera: Chrysomelidae: Bruchinae) was sequenced using Illumina’s HiSeq2000 platform. The mitogenome is a double-stranded circular molecule of 17,809 bp in length with 21 transfer RNA genes, 13 protein-coding genes, and two ribosomal RNA genes as in other insects. Specially, there is a 2008 bp-inserted segment between ND2 and tRNA-Trp from 1180 to 3187, which cannot be aligned to any known gene of mitogenomes. To estimate the taxonomic status of Bruchinae, total 17 species from eight subfamilies of Chrysomelidae were selected as ingroups and three species of Lamiinae as outgroups for phylogenetic analysis based on mitogenome. The results showed that three major lineages were formed, including a basal ‘Eumolpine’ clade (Cassidinae, Eumolpinae, Cryptocephalinae, Clytrinae), ‘'Criocerine’ clade (Criocerinae, Bruchinae) and ‘Chrysomeline’ clade (Chrysomelinae, Galerucinae s. l.). Bruchinae showed more closed relationship with Criocerinae than other subfamilies. More thorough taxon sampling will be needed to well understand the relationship in Chrysomelidae.

*Callosobruchus maculates* (F.) (Coleoptera: Chrysomelidae: Bruchinae) is an important insect pest of cowpea in the store causing considerable damage to the grains. During larval stages, it causes substantial quantitative and qualitative losses (50–90%) manifested by seed perforation and reductions in weight, mark value, and germination ability of seeds (Ofuya and Osadahun 2005; Brisibe et al. 2011; Akami et al. 2016). The beetle most likely originated in West Africa and moved around the globe with the trade of legumes and other crops (Tran and Credland 1995). Now, this pest of stored legumes has a cosmopolitan distribution.

Traditionally, bean weevil was considered a separate family within Chrysomelidae (Crowson 1955; Kingsolver 1996; Reid 1996; Verma and Saxena 1996; Duckett 1997; Lingafelter and Pakaluk 1997; Verma 1998), but the group was demoted to subfamily rank in several phylogenetic studies based on molecular data (Reid 1995, 2000; Farrell and Sequeira 2004; Gómez-Zurita et al. 2008; Bocak et al. 2014; Haddad and Mckenna 2016). According to Bouchard et al. (2011), Chrysomelidae contains 13 subfamilies: Sagrinae, Bruchinae, Donaciinae, Criocerinae, Cassidinae, Chrysomelinae, Galerucinae, Lamprosomatinae, Cryptocephalinae, Eumolpinae, Spilopyrinae, Synetinae, and Protoscelidinae. Recently, with the development of next-generation sequencing, a large-scale mitogenome has been widely used to resolve the phylogeny and evolution of organisms across the tree of Coleoptera (Timmermans et al. 2010; Crampton-Platt et al. 2015; Gomez-Rodriguez et al. 2015; Timmermans et al. 2016; Haddad and Mckenna 2016; Nie et al. 2017). In this study, the complete mitogenome of *C. maculates* was sequenced by next-generation sequencing technology to estimate the taxonomic status of Bruchinae.

The specimens used in this study were intercepted in imported *Vigna unguiculata* from Nigeria and deposited in the plant laboratory of Beijing Inspection and Quarantine Testing Center. Genomic DNA was extracted by TIANprep Midi Plasmid kit (TIANGEN, Beijing, China) and then sequenced using Illumina’s HiSeq2000 platform (Illumina, San Diego, CA) with 200 bp insert size and a pair-end 100 bp sequencing strategy. The sequence reads were first filtered by the programs following Zhou et al. (2013) and then the remaining high-quality reads were assembled using SOAPdenovo-Trans (Xie et al. 2014). The annotations of genes were done by Geneious 8.0.5 software (Kearse et al. 2012) and tRNAscan-SE 1.21 (Schattner et al. 2005). In order to confirm the insert gene between ND2 and tRNA-Trp from 1180 to 3187, we sequenced this species twice sampling two specimens using Illumina’s HiSeq2000 platform and Illumina’s HiSeq2500 platform.

The complete mitochondrial genome (mitogenome) of *C. maculates* is a double-stranded circular molecule of 17,809 bp in length (GenBank accession number: MF960125), with 21 transfer RNA genes (tRNA-Gln lost), 13 protein-coding genes, and two ribosomal RNA genes as in other insects. The overall base composition is A: 45.5%, T: 34.8%, C: 13.6%, and G: 6.1%, with a much higher A + T content. Specially, there is 2008 bp inserted genes between ND2 and tRNA-Trp from 1180 to 3187, which cannot be aligned to any known gene of mitogenome. We had blasted and tried to annotate the insert gene. However, there was no gene matching this fragment. It will be worthy to add more mitogenomes of other species of Bruchinae to explore the potential function of inserted genes and why the mitogenome lost tRNA-Gln.

The phylogenetic tree was reconstructed to estimate the status of Bruchinae in Chrysomelidae. All available mitogenomes of subfamilies of Chrysomelidae were downloaded from Genbank. The acceptable sequences including 13 protein-coding genes and longer than 10K bp were kept. Total 17 species from eight subfamilies (accession numbers: JX220988, JX412753, JX412756, HQ232809, JX412804, MF960125, JX412832, AF467886, JX412769, JX220992.1, MF946616, KF669870, KF658070, NC_028332, MF960109, MF960113, MF960117) were selected as ingroups and three species of Lamiinae (accession numbers: DQ768215, NC_022671, FJ424074) was selected as outgroups. The combined data set of 13 protein-coding gene (PCGs) were aligned with TransAlign (Bininda-Emonds 2005). The data aligned from 13PCGs were concatenated with Sequence Matrix v.1.7.8 (Vaidya et al. 2011). The Bayesian phylogenetic inference was performed using MrBayes v.3.2 (Ronquist et al. 2012) based on the combined data set of 13 PCGs. Data were partitioned according to loci of 13 PCGs. The MCMC search was conducted for 1,000,000 generations, and sampling was done every 100 generations until the average standard deviation of split frequencies was below 0.01. The first 25% of trees were discarded as ‘burn-in’ and posterior probabilities were estimated for each node.

Phylogenetic analyses (Figure 1) showed that three major lineages were formed, including a basal ‘Eumolpine’ clade (Cassidinae, Eumolpinae, Cryptocephalinae, Clytrinae), ‘Criocerine’ clade (Criocerinae, Bruchinae), and ‘Chrysomeline’ clade (Chrysomelinae, Galerucinae s. l.). Bruchinae showed more closed relationship with Criocerinae than other subfamilies. However, more thorough taxon sampling will be needed to well understand the relationship in Chrysomelidae because mitogenomes of only eight subfamilies were involved in the present study.

**Figure 1.**
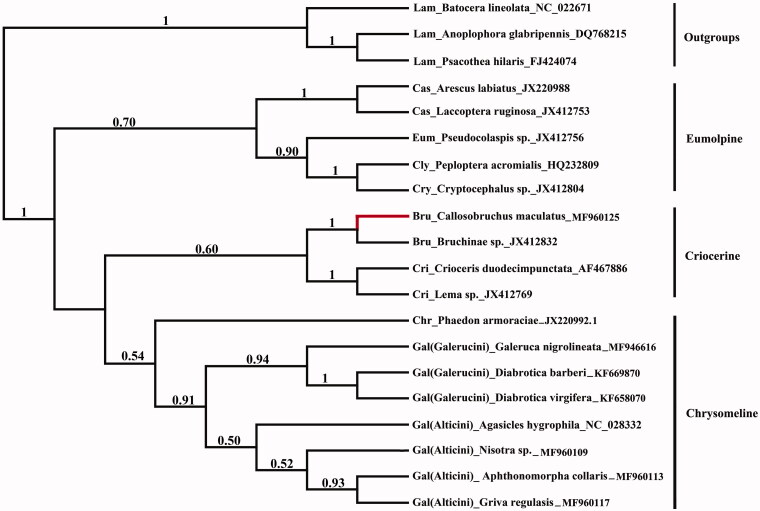
The Bayesian tree based on 13 PCGs combined data sets. Numbers on nodes indicate Bayesian posterior probabilities. Gray branch is the new data in this study.
